# Relationship of oxidative stress in the resistance to imatinib in Tunisian patients with chronic myeloid leukemia: A retrospective study

**DOI:** 10.1002/jcla.23050

**Published:** 2019-10-16

**Authors:** Mariam Ammar, Lobna Ben Mahmoud, Moez Medhaffar, Hanen Ghozzi, Zouheir Sahnoun, Ahmed Hakim, Malek Mseddi, Moez Elloumi, Khaled Zeghal

**Affiliations:** ^1^ Department of Pharmacology Faculty of Medicine University of Sfax Sfax Tunisia; ^2^ Faculty of Sciences of Sfax University of Sfax Sfax Tunisia; ^3^ Department of Hematology Hedi Chaker Hospital University of Sfax Sfax Tunisia

**Keywords:** chronic myeloid leukemia, imatinib resistance, oxidative stress

## Abstract

**Background:**

This work aimed to evaluate oxidative stress in chronic myeloid leukemia (CML) patients treated with tunisian (IM) vs controls and in CML patients with resistance to IM vs patients without resistance to IM.

**Methods:**

The study included 40 CML patients and 34 controls. Of 40 patients with CML, 26 patients were developed in resistance to IM. The oxidant/antioxidant markers were evaluated by spectrophotometric methods for all used samples.

**Results:**

For CML patients, increased malondialdehyde (MDA) and advanced oxidation protein products (AOPP) levels were found compared to controls (*P* < .001; *P* = .01). Higher catalase (CAT) activity (*P* = .048) and lower superoxide dismutase (SOD) and glutathione peroxidase (GPx) activities, reduced Glutathione (GSH) and vitamin C levels were found in CML patients (*P* < .001). The comparison between the resistant vs no‐resistant CML patients revealed higher MDA level (*P* = .02) and CAT and SOD activities in IM‐resistant patients (*P* = .04, *P* = .03). GPx activity was reduced (*P* = .04). Furthermore, increased mean ratio of MDA/GSH, MDA/GPx, and SOD/(GPx + CAT) was found in IM‐resistant patients as compared with no‐resistant (*P* = .01, *P* = .01, *P* = .035). The mean ratio of GPx/GSH in the IM‐resistant CML patients was lower than in IM no‐resistant one (*P* = .039). For IM‐resistant patients, we found negative correlation between MDA level and the ratio SOD/(CAT + GPx) (*r *= −0.46, *P* = .002); and positive correlation between SOD and (CAT + GPx) activities (*r* = 0.38, *P* = .06) and between GSH level and GPx activity (*r* = 0.53, *P* = .01).

**Conclusions:**

Our results have shown a highly disturbed oxidative profile in IM‐resistant CML patients as compared to no‐resistant. The H_2_O_2_ has a key role in the resistance to IM treatment.

## Graphical abstract

High malondialdehyde and advanced oxidation of protein products levels were evidenced in chronic myeloid leukemia. Disturbed enzymatic and no enzymatic defenses were found in plasma of chronic myeloid leukemia patients. Patients with resistance to imatinib revealed more pronounced oxidant/antioxidant markers than no‐resistant chronic myeloid leukemia patients. Hydrogen peroxide was incriminated in the disturbed oxidative profile in chronic myeloid leukemia patients with resistance to imatinib.

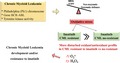

## 1 INTRODUCTION

Chronic myeloid leukemia (CML) is a malignant myeloproliferative disorder caused by the BCR‐ABL dysfunctional protein.^1^, ^2^ The development of inhibitors specifically of the BCR‐ABL tyrosine kinase activity as a therapeutic agent has revolutionized CML treatment.^3^, ^4^, ^5^ Imatinib mesylate (IM) is the first generation of tyrosine kinase inhibitors (TKIs) that inhibit the activity of the BCR‐ABL tyrosine kinase by blocking the ATP‐binding site and consequently inducts apoptosis in the CML cells.^6^, ^7^, ^8^ Notwithstanding the excellent results obtained with IM for the therapy of CML, a minority of CML patients acquired resistance to IM, which has become an emerging problem resolved only in part by second and third generations of TKIs.^9^, ^10^

Various mechanisms of IM resistance were identified, including overexpression of the BCR‐ABL gene, mutations in the BCR‐ABL kinase domain and genetic variations and/or altered expression of IM gene transporters.^11^, ^12^, ^13^, ^14^ Among them, the reactivation of BCR‐ABL protein by mutations in the kinase domain, such as T315I mutation, was highly documented as one of the most prevalent mechanisms leading to IM resistance.^14^ H Zhang et al demonstrated that the oxidation of redox‐sensitive cysteine residues of BCR‐ABL protein by reactive oxygen species (ROS) can lead to a change in the three‐dimensional structure of the ABL kinase domain of this oncoprotein that is the domain of IM.^3^ The precise mechanisms underlying the resistance to IM therapy remain largely elusive and controversial. This study opens new perspectives for future works to solve the relapse issue of CML patients treated with IM.

The impact of oxidative stress on CML improvement is not clear. Previous investigations have shown that the transformation of BCR‐ABL oncogene can promote the generation of ROS and redox imbalance.^15^, ^16^, ^17^ However, others have incriminated the overproduction of ROS in the CML cells and exogenous ROS in the development of CML as well as in the resistance to IM. 8, 9, 18, 19, 20, 21, 22, 23, 24, 25Therefore, a systemic analysis of the markers of oxidative stress in plasma of Tunisian patients with CML has been undertaken. The present study was conducted to evaluate the markers of oxidative stress: (a) in CML patients treated with TKI and healthy subjects in order to understand the association of ROS in CML, and (b) in CML patients IM‐resistants and IM no‐resistants to explain the mechanism of IM resistance.

## 2 PATIENTS AND METHODS

### 2.1 Ethics statement

The experimental protocol was established in accordance with the guidelines of the Declaration of Helsinki, and informed consent was obtained from patients.

### 2.2 Patients

Forty CML patients (26 men and 14 women) were recruited from the Hematology Department of the Hedi Chaker University Hospital, Sfax, Tunisia, from April 2017 through March 2018. Patients suffering from any another hematological illness, taking vitamins with antioxidant activity or with BCR‐ABL1 gene mutations were excluded. According to the standard hematological, cytogenetic, and molecular European Leukemia Network (ELN 2016) criteria,^26^  CML patients were classified into two groups: IM no‐resistant CML patients (n = 14) who attained a major molecular response (MMR) at 1 year; and IM‐resistant CML patients (n = 26), who did not show MMR during 1 year of IM treatment. Patient's demographic and biological data (complete blood count and spleen volume), blood disorders, and therapy failure were obtained from hospital records.

The BCR‐ABL gene mutation testing was performed exclusively on patients who showed IM resistance.

Thirty‐four healthy subjects (25 men and 9 women) were collected from the Regional Blood Transfusion Center, and those with any systemic or chronic disease were excluded from this study.

### 2.3 Plasma samples

Blood was collected by venepuncture from CML patients and controls in tubes comprising ethylenediaminetetraacetic acid (EDTA). The plasma layer was separated by centrifugation at 4042 *g* for 10 minutes. Samples were immediately frozen and stored at −80°C in a small aliquot until analysis.

### 2.4 Protein concentration

The protein content was assessed by the Bradford assay^27^ using bovine serum albumin (BSA) as a standard. The protocol consisted in adding 5 µL of plasma (1:100) with 295 µL of Bradford reagent. After gentle vortex, the samples were measured at 595 nm. Protein concentration was expressed as mg/mL.

### 2.5 Malondialdehyde level

The lipid peroxidation in the plasma was evaluated by malondialdehyde (MDA) level, using thiobarbituric acid reactive species (TBARS) assay.^28^ The plasma was heated with the reagent (containing 0.8% thiobarbituric acid, 15% trichloroacetic acid, and 25% hydrochloric acid) during 15 minutes at 95°C. After cooling, the sample was centrifuged and measured spectrophotometrically at 532 nm. Plasmatic MDA level was expressed as n moles MDA/mg of total protein.

### 2.6 Advanced oxidation protein products level

The advanced oxidation protein products (AOPP) level was measured according to the method described by Kayali et al^29^ Briefly, 96 µL of plasma samples diluted to 1:3 in phosphate‐buffered saline (PBS; 0.1 mol/L) were treated with 20 µL of potassium iodide (1.16 mol/L). Followed 2 minutes later, 40 μL of glacial acetic acid was added. The optical density was then read immediately at 340 nm. The concentration of AOPP was expressed as n moles AOPP/mg of protein using the extinction coefficient ε340 = 261 mmol/L^−1^ cm^−1^.

### 2.7 Ascorbic acid level

The ascorbic acid (vitamin C) level was determined based on the method of Jacota and Dani.^30^ To 100 µL of plasma, 400 µL of 10% trichloroacetic acid was added. After cooling, the sample was centrifuged at 4000 rpm for 5 minutes. The extract was diluted to 1:2 and then added to 200 µL of diluted Folin reagent. After 10 minutes, the absorption maximum of the blue colored complex developed by the interaction of ascorbic acid with Folin reagent was read at a wavelength of 769 nm. The results were expressed as ng of ascorbic acid/ mg of total protein.

### 2.8 Reduced glutathione level

The glutathione (GSH) level was determined by the method of Ellman^31^ based on the oxidation of the GSH by Ellman's reagent (sulfhydryl reagent 5,5′‐dithio‐bis(2‐nitrobenzoic acid) [DTNB]) for generating the yellow derivative 5′‐thio‐2‐nitrobenzoic acid (TNB).

After deproteinizing plasma by 4% sulfosalicylic acid (v/v), the extract was added to 160 µL of PBS (0.1 mol/L) and 64 µL of Ellman's reagent. The sample was rapidly covered with aluminum foil and then incubated for 15 minutes. This latter was measurable spectrophotometrically at a wavelength of 412 nm. The plasmatic GSH level was expressed as n moles GSH/mg of total protein.

### 2.9 Glutathione peroxidase activity

The activity of glutathione peroxidase (GPx) was performed by the method of Flohé and Günzler^32^ based on GSH oxidation by GPx in the presence of DTNB. The experimental protocol consisted in incubating a mixture containing 200 µL of plasma, 100 µL of PBS (0.1 mol/L), and 200 µL of GSH (4 mmol/L) at 37°C for 15 minutes. The reaction was initiated by adding 500 µL of hydrogen peroxide (H_2_O_2_) (5 mmol/L). One min later, this reaction was stopped by 1 mL of 5% trichloroacetic acid. After centrifugation, the extract was incubated with 1 mL of PBS (0.1 mol/L) and 500 µL of DTNB (10 mmol/L) in the dark for 5 minutes. The optical density was determined at 412 nm. GPx activity was expressed as n moles of disappeared GSH/min/mg of total proteins.

### 2.10 Catalase activity

The activity of Catalase (CAT) was quantified by the procedure of Aebi based on the decomposition rate of H_2_O_2_.^33^ This reaction was expressed as U/mg of protein by using wavelength 240 nm with molar extinction coefficient ε_240_ = 43.1 M^−1^ cm^−1^.

The protocol consisted in disposing 285 µL of PBS (0.1 mol/L) in two spectrophotometer cuvettes and adding 15 µL of H_2_O_2_ except to an only one cuvette. After measurement at 240 nm, 5 µL of the plasma was added in the first and second cuvette. The optical density was also read immediately at 240 nm.

### 2.11 Superoxide dismutase activity

The activity of Superoxide dismutase (SOD) was determined by measuring its capacity to inhibit the photochemical reduction of nitroblue tetrazolium (NBT) according to Beauchamp and Fridovich.^34^ The experimental protocol consisted in incubating a mixture containing 25 µL of plasma (1:50), 921 µL of PBS (0.1 mol/L), 500 µL of EDTA‐Na_2_‐methionine (2.96 mmol/L), 42 µl of NBT (2.64 mmol/L), and 11 µL of riboflavin (0.26 mmol/L) in light for 20 minutes. The reduction of NBT by O_2_^•−^ to blue colored formazan was followed at 580 nm. One unit of SOD activity is described as the amount of enzyme that inhibits the rate of NBT photoreduction rate by 50% under the specified conditions. The SOD activity was expressed as U/mg of protein.

### 2.12 Statistical analysis

Statistical differences between groups were performed using the unpaired *t* test. The correlation study was assessed by the Pearson correlation test. The significance was obtained for a *P*‐value < .05. All results are expressed as mean ± standard error mean (SEM). The statistical analyses and the figures were performed using GraphPad Prism 6.

## 3 RESULTS

### 3.1 Patients' characteristics

The study cohort was composed of 40 patients with CML (26 M/14 F; the mean age was 49 ± 9.2 years). According to ELN criteria 2016,^26^ 14 patients were IM responders, whereas 26 patients showed IM‐resistant phenotype. Among these IM‐resistant CML patients, 23 patients failed to achieve an optimal response defining primary resistance and three lost their initial response and developed a secondary resistance. The *BCR‐ABL1* gene mutation testing was performed exclusively on patients who showed IM resistance. This test indicated no mutation of the BCR‐ABL kinase domain in these subjects.

Each patient was described by levels of white blood cell (WBC), neutrophils, platelets and hemoglobin, and by the spleen volume. As shown in Table 1, levels of WBC and neutrophils were significantly higher for IM‐resistant patients in comparison to IM no‐resistant patients (*P* = .005 and *P* = .01, respectively). In addition, for IM‐resistant patients, the volume of the spleen was greater than for IM no‐resistant patients (*P* = .02) (Table 1).

**Table 1:** Comparison of biological characteristics between IM no‐resistant CML patients and IM‐resistant CML patients

Biological characteristics	No‐resistant (n = 14)	Resistant (n = 26)	*P*‐value
WBC (10^3^/mm^3^)	6.20 ± 1.19	13 ± 1.03	**.005**
Neutrophils (10^3^g/l)	3.06 ± 1.74	8.53 ± 1.33	**.01**
Platelets (10^3^/mm^3^)	209 ± 1.19	255.5 ± 1.03	.372
Hb (g/dL)	12.67 ± 1.19	12.19 ± 1.03	.494
Spleen volume (cm)	5.72 ± 1.27	11.5 ± 1.09	**.02**

Note

Data presented as mean ± SD and *t* test applied for the comparisons.

Abbreviations: Hb, hemoglobin; WBC, white blood cell.

Bold values show P<0.05, significant

Thirty‐four healthy volunteers (25 M/9 F; mean age 42.14 ± 9.04) were also included as a control group in the study. No significant differences were found for these characteristics between controls and CML patient groups (*P* > .05).

### 3.2 Evaluation of oxidative profile in CML patients

#### 3.2.1 Oxidation markers

**Figure 1:**
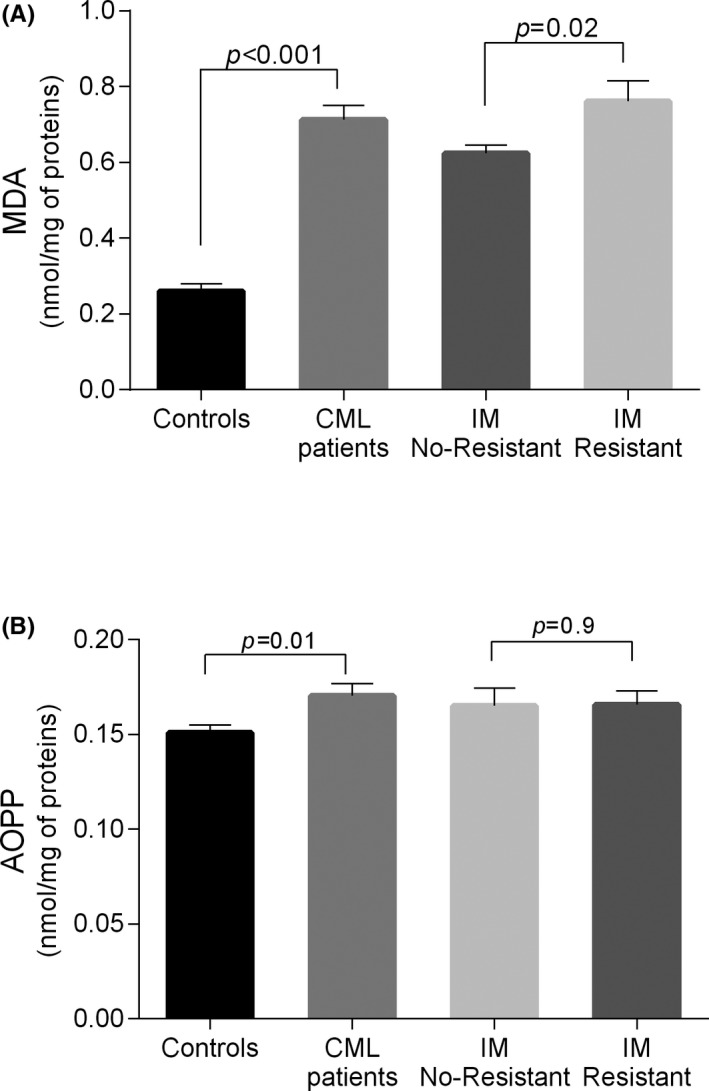
Oxidant plasma levels in CML patients vs controls and IM no‐resistant vs IM‐resistant CML patients. Each column represents mean ± SEM. CML, chronic myeloid leukemia; IM, imatinib mesylate

Two markers of oxidative stress were evaluated in plasma of CML patients and controls. The results showed significant increased MDA as well as AOPP levels in plasma of CML patients compared to controls (*P* < .001 and *P* = .01 respectively) Figure 1A, B respectively).

#### 3.2.2 Antioxidant defense

**Figure 2:**
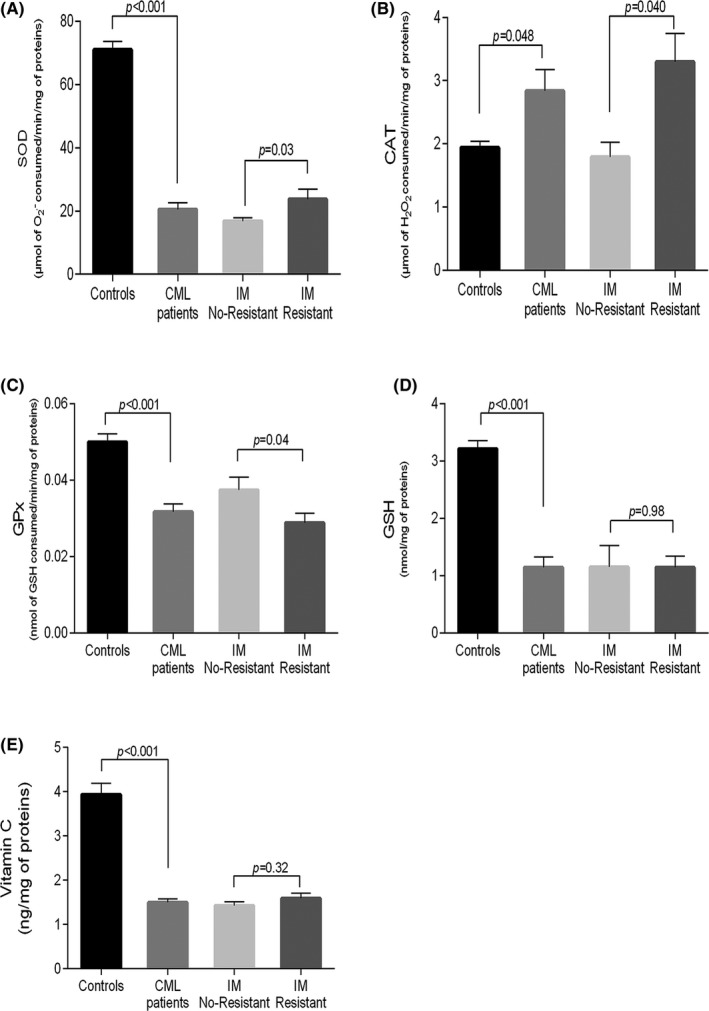
Antioxidant enzymatic and non‐enzymatic defenses in CML patients vs controls and IM. No‐resistant vs IM‐resistant CML patients. Each column represents mean ± SEM. CML, chronic myeloid leukemia; IM, imatinib mesylate

The CAT activity was higher in CML patients’ plasma compared to the control group (*P* = .048; Figure 2B). In contrast, SOD and GPx activities were found to be lower in plasma of CML patients as compared to controls (*P* < .001; Figure 2A, C respectively). Moreover, decreased levels of GSH and vitamin C were found in patients in comparison to controls (*P* < .001; Figure 2D, E respectively).

### 3.3 Comparison of oxidative profile of IM no‐resistant patients vs IM‐resistant patients

#### 3.3.1 Oxidation markers

The comparison between the IM no‐resistant and IM‐resistant patients showed a higher rate of MAD in IM‐resistant (*P* = .02; Figure 1A). However, no difference was found for the AOPP level (Figure 1B).

#### 3.3.2 Antioxidant defense

The comparison by the unpaired *t* test revealed that CAT and SOD activities were increased in IM‐resistant patients with regard to the IM no‐resistant patients (*P* = .04, *P* = .03 respectively; Figure 2A, B respectively). In contrast, GPx activity was reduced in IM‐resistant patients when compared to IM no‐resistant one (*P* = .04; Figure 2C). No significant differences were observed between IM‐resistant and IM no‐resistant patients concerning the GSH and the vitamin C levels (*P* = .98, *P* = .32, respectively; Figure 2D, E, respectively).

Our study showed significant increase in the mean ratio of MDA/GSH, MDA/GPx, SOD/GPx, and SOD/ (GPx + CAT) in IM‐resistant patients when compared with no‐resistant (*P* = .01, *P* = .01, *P* = .005, *P* = .035, respectively; Table 2). The mean ratio of GPx/GSH in IM‐resistant CML patients was lower than that of IM no‐resistant one (*P* = .039; Table 2).

**Table 2:** Comparison of the ratio oxidant/antioxidant between IM no‐resistant and IM‐ resistant CML patients

	No‐resistant (n = 14)	Resistant (n = 26)	*P*‐value
MDA/GSH	0.38 ± 0.03	1.28 ± 0.17	**.01**
MDA/GPx	20.6 ± 1.8	29.16 ± 2.3	**.01**
MDA/CAT	3.8 10^−4^ ± 6.4 10^−5^	3.7 10^−4^ ± 4.7 10^−5^	.925
MDA/SOD	4.12 10^−5^ ± 3.2 10^−6^	4.2 10^−5^ ± 4.9 10^−6^	.917
GPx/GSH	0.1 ± 0.04	0.03 ± 0.0	**.039**
SOD/GPx	4.8 10^5^ ± 4.9 10^4^	6.7 10^5^ ± 3.8 10^4^	**.005**
SOD/(GPx + CAT)	8.18 ± 1.03	13.06 ± 2.3	**.035**

Note

Each result represents mean± SD Student's *t* test.

Bold values show P<0.05, significant

### 3.4 Correlation studies

**Figure 3:**
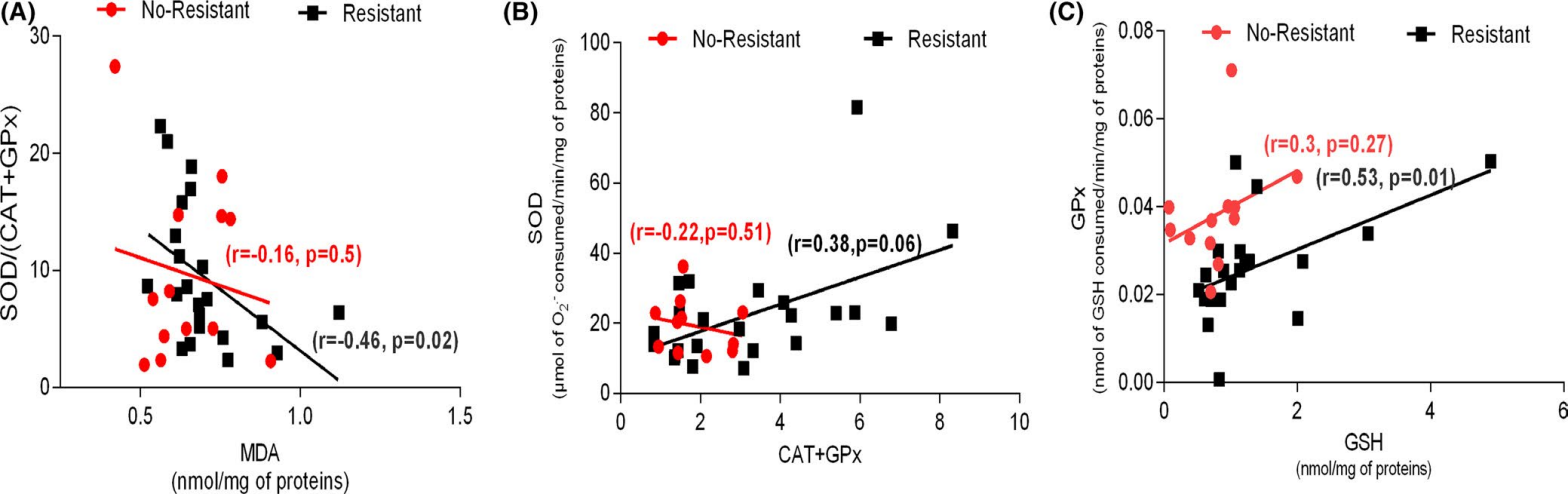
A, Correlation between MDA level and the ratio *R* = SOD/(GPx + CAT; activities of enzymes) in CML patients IM no‐resistant and CML patients IM‐resistant. B, Correlation between CAT + GPx activities and SOD activity in CML patients IM no‐resistant and CML patients IM‐resistant. C, Correlation between GSH level and GPx activity in CML patients IM no‐resistant and CML patients IM‐resistant. CML, chronic myeloid leukemia; IM, imatinib mesylate

The correlation study was conducted between oxidative stress parameters for the two subgroup IM no‐resistant and IM‐resistant CML patients. For IM‐resistant patients, negative correlation was observed between MDA level and the ratio SOD/(CAT + GPx) (*r* = −0.46, *P* = .002; Figure 3A). However, the correlations between SOD and (CAT + GPx) activities and GSH level and GPx activity were found to be positive (*r* = 0.38, *P* = .06; *r* = 0.53, *P* = .01, respectively; Figure 3B, C). For IM no‐resistant patients, no significant correlations were noted (Figure 3).

## 4 DISCUSSION

It is well known that overproduction of ROS such as superoxide radicals (O_2_^•−^), hydrogen peroxide (H_2_O_2_), and the highly reactive hydroxyl radicals (OH^•^) leads to the oxidative damage of lipids, proteins, and DNA.^35^, ^36^ If not repaired DNA damage may bring mutagenesis and cause the onset or the development of different types of cancers,^36^, ^37^, ^38^, ^39^ high ROS level was particularly incriminated in the resistance to BCR/ABL kinase inhibitors established in experimental cell lines. 6, 8, 40 Our study was conducted to comprehend the mechanisms of resistance to IM order to find therapeutic solutions that may help prevent a relapse from this drug in CML. In this review, we have mainly focused on the mechanism of resistance to IM induced by oxidative stress in CML. To the best of our knowledge, this is the first Tunisian study to measure the variation of the markers of oxidative stress in CML patients.

Our findings revealed for the first time high rates of MDA and AOPP levels associated with disturbed antioxidant markers in plasma of CML patients compared to controls. These results confirmed the previous reports showing increased levels of MDA and carbonyl group in plasma of AML and CML patients.^37^, ^41^, ^42^, ^43^ Antioxidant enzyme activities investigation showed decreased SOD activity in CML patients. This result is in accord with a previous work by Al‐Rasheed et al on albino rats where the liver tissue of rats treated with Gleevec displayed reduced SOD activity compared to a control group.^44^ Several investigators have documented the low SOD activity in many other types of leukemia such as acute myeloid leukemia (AML) and chronic lymphocytic leukemia (CLL) and opted for an inhibition of the enzyme by the high rate of ROS.^45^, ^46^ Indeed, it was shown that significant amounts of (O_2_^•−^) were generated by leukocytes isolated from AML patients with reduced SOD activity.^46^ The association of the elevated concentration of MDA and the high level of (O_2_^•−^) produced by the leukocytes of AML patients was likely to be at the origin of the SOD inhibition.^46^ Moreover, the findings of zhou et al indicated that CLL cells produced endogenous (O_2_^•−^) and that higher level of superoxide was observed in leukemic cells of patients treated with chemotherapy. Hence, the authors predicted that cells owning high (O_2_^•−^) level are more vulnerable to SOD inhibition.^45^ The SOD is one of the main antioxidant enzymes since it catalyzes the dismutation of the highly reactive (O_2_^•−^) to O_2_ and H_2_O_2_, a less ROS. The H_2_O_2_ is then converted to non‐toxic molecules by other enzymes, such as catalase and GPx.^46^ Interestingly, increased activity of the CAT enzyme was observed in CML patients. These data suggest that the high enzyme activity was probably a compensatory response to H_2_O_2_ predominantly derived from cancer cells rather than the dismutation of the (O_2_^•−^) by the SOD enzyme. Considerable evidence has built up from experiments on cancer cell lines to support the notion that cancer cells are well considered as sources of ROS production.^45^, ^47^, ^48^ Concerning GPx activity and GSH level, the comparison between the CML patients and the controls displayed significant decrease for both markers. These findings are in line with a previous comparative study conducted on plasma of AML and CML patients showing reduced level of glutathione.^37^ In addition, the same result was noted in a recent work conducted on rats.^44^ GPx enzyme reduces the H_2_O_2_ and the organic peroxides while oxidizing the GSH. The oxidized glutathione, GSSG, is reduced back to GSH by the glutathione reductase enzyme (GRx) in NADPH presence.^49^, ^50^ Furthermore, the evaluation of the vitamin C level showed low level in CML patients, which reinforces the disturbance in the oxidant/antioxidant status observed in these patients.

To gain more insight into the interrelationship between the disturbed oxidative profiles evidenced in CML patients and the resistance to IM treatment, the oxidant/antioxidant statuses were investigated, in a comparative way, in both subgroup IM‐resistant and IM no‐resistant CML patients. Our results have clearly shown significant differences in the MDA level and the antioxidant enzyme activities with more pronounced oxidative stress in IM‐resistant patients compared to no‐resistant ones. This was confirmed by the negative correlation between the MDA level and the antioxidant enzyme ratio activities; and by the increased ratio MDA/GPx and MDA/GSH noted in IM‐resistant CML patients. This result is in agreement with the study by Rizwan et al showing higher MDA levels in CML patients with chronic phase who progressed to the accelerated phase vs CML patients with chronic phase without progression to the accelerated phase.^42^ Our results concerning the antioxidant enzyme activities demonstrate unprecedently that IM‐resistant CML patients exhibited the lowest GPx activity compared to the no‐resistant ones. The activity of this enzyme correlates positively with the GSH level in IM‐resistant patients with significant results. In addition, the MDA/GPx and MDA/GSH ratios were higher in IM‐resistant patients. Given all these findings, we suggest that the decrease of the GPx activity was probably due to the low GSH level since it represents its coenzyme. Interestingly, the SOD and CAT activities were increased in IM‐resistant vs IM no‐resistant CML patients. Furthermore, increased ratio SOD/ (CAT + GPx) as well as positive correlations between SOD and (CAT + GPx) activities were observed in IM‐resistant patients. The main inference we can draw from these findings is that in the case of resistance to IM, CML patients have elevated levels of (O_2_^•−^) and particularly H_2_O_2_. The latter could be generated following the disturbed oxidative profile in IM‐resistant patients but also as a result of the dismutation of the (O_2_^•−^) to the H_2_O_2_ and O_2_ by the SOD enzyme.^51^, ^52^ These ROS represent the substrates of these two enzymes, and their presence at relatively higher levels is a stimulating factor for enzymatic activities. Indeed, the CAT enzyme was the major source of protection against high levels of peroxides under severe oxidative stress, whereas GPx becomes more significant in low levels of H_2_O_2_.^53^ Therefore, we suggest that H_2_O_2_ plays a crucial role in the case of IM resistance and CML patients with IM treatment presenting increased CAT and SOD activities are more likely to evolve to the resistance state. As a matter of fact, contrary to most ROS, H_2_O_2_ has a long half‐life and can cause DNA damage even at distant sites.^54^In the CML IM resistance case, the mutation T315I was frequently observed and even the second‐generation BCR‐ABL inhibitors remain ineffective.^48^ Among the proposed approaches is the use of oxidative stress in overcoming IM resistance. The study conducted on CML model cells showed that cells with T315I mutation displayed greater extent of H_2_O_2_‐induced mtDNA damage than their imatinib‐sensitive counterparts.^6^ Other investigations on CLL cells and trying the use of oxidative stress in the anti‐leukemia therapy, proposed 2‐Methoxyestradiol as a new anticancer agent currently in clinical trials that inhibit the SOD activity and induces apoptosis in leukemia cells through the accumulation of (O_2_^•−^).^45^

Further large‐scale investigations are mandatory to elucidate the fact that targeting ROS level could be a novel approach to overcome IM resistance.

## 5 CONCLUSIONS

So far, the research we carried out has been an attempt to explore the relationship of oxidative stress in the resistance to IM in Tunisian patients with CML. We demonstrate the presence of oxidative stress in plasma of CML patients characterized by high levels of MDA and a disturbance in the antioxidant activities. The study provides evidence for more pronounced disturbance in the oxidant‐antioxidant in IM‐resistant CML patients compared to no‐resistant ones. It seems that O_2_^•−^ and particularly H_2_O_2_ have a key role in the resistance to IM treatment, which could contribute to the development and/or the progression to more severe conditions.
